# Varicella vaccination coverage of children under two years of age in Germany

**DOI:** 10.1186/1471-2458-10-502

**Published:** 2010-08-19

**Authors:** Annicka M Reuss, Marcel Feig, Lutz Kappelmayer, Anette Siedler, Tim Eckmanns, Gabriele Poggensee

**Affiliations:** 1 Robert Koch Institute, DGZ-Ring 1, 13086 Berlin, Germany; 2 Medizinische Fakultät Charité - Universitätsmedizin Berlin, Charitéplatz 1, 10117 Berlin, Germany

## Abstract

**Background:**

Since July 2004, routine varicella vaccination is recommended by the German Standing Vaccination Committee in Germany. Health Insurance Funds started to cover vaccination costs at different time points between 2004 and 2006 in the Federal States. Nationwide representative data on vaccination coverage against varicella of children under two years of age are not available. We aimed to determine varicella vaccination coverage in statutory health insured children under two years of age in twelve German Federal States using data from associations of statutory health insurance physicians (ASHIPs), in order to investigate the acceptance of the recommended routine varicella vaccination programme.

**Methods:**

We analysed data on varicella vaccination from 13 of 17 ASHIPs of the years 2004 to 2007. The study population consisted of all statutory health insured children under two years of age born in 2004 (cohort 2004) or 2005 (cohort 2005) in one of the studied regions. Vaccination coverage was determined by the number of children vaccinated under 2 years of age within the study population.

**Results:**

Varicella vaccination coverage of children under two years of age with either one dose of the monovalent varicella vaccine or two doses of the measles, mumps, rubella, and varicella vaccine increased from 34% (cohort 2004) to 51% (cohort 2005) in the studied regions (p < 0.001). More than half of the vaccinated children of cohort 2004 and two third of cohort 2005 were immunised at the recommended age 11 to 14 months. The level of vaccination coverage of cohort 2004 was significantly associated with the delay in introduction of cost coverage since the recommendation of varicella vaccination (p < 0.001).

**Conclusions:**

Our study shows increasing varicella vaccination coverage of young children, indicating a growing acceptance of the routine varicella vaccination programme by the parents and physicians. We recommend further monitoring of vaccination coverage using data from ASHIPs to investigate acceptance of the routine vaccination programmes over time.

## Background

In May 2004, a consensus statement on varicella vaccination policy was published by the European Working group on Varicella (Eurovar) [1]. Routine varicella vaccination for children during early childhood and catch-up vaccination for adolescents and adults without history of varicella were recommended for countries where high vaccination coverage could be anticipated. Vaccination policies differ considerably between countries [2,3]. In the United States, varicella vaccination is part of the childhood immunization schedule since 1996 and the vaccination programme had been proven to effectively reduce varicella incidence and hospitalization rates [4,5]. In Europe, vaccination of children or adolescents against varicella is included in national immunization schedules of eight countries. Target groups of vaccination programmes are mainly high risk groups or children with no history of varicella disease e.g. in Austria, Spain and Switzerland.

Germany is one of the countries where routine vaccination of all children in early childhood is recommended as well as e.g. Latvia, Greece and three regions of Italy [6,7]. Routine varicella vaccination was recommended by the German Standing Committee on Vaccination (STIKO) in 2004 after studies had shown a higher rate of complications in infants and young children in Germany than previously assumed [8-10]. According to the national immunization schedule 2004, children should be immunised with one dose of varicella vaccine at age 11 to 14 months. Unvaccinated older children and adolescents without history of varicella were also scheduled for vaccination [8]. Children are usually vaccinated by their paediatrician. Since vaccinations are voluntary in Germany, physicians play a major role in advising parents on vaccination programmes and in reminding them of upcoming vaccinations for their children according to the schedule [11]. Aim of the routine varicella vaccination programme was to reduce varicella morbidity, including complications and hospitalisations [8].

Health Insurance Funds started to cover costs for varicella vaccination at different time points between 2004 and 2006 in the Federal States, depending on agreements with the ASHIPs. Since 2007, Health Insurance Funds are obliged by law to cover costs for vaccinations recommended by the STIKO and approved by the Federal Joint Committee [12].

Given that varicella is not a notifiable disease in Germany, a nationwide sentinel surveillance system was established by the Working Group on Measles and Varicella (AGMV) in 2005 to monitor trends in the number of varicella vaccinations and varicella cases [13]. The sentinel system is based on reports from a sample of voluntarily participating physicians which treat an undefined proportion of the population because patients are free to choose their physician. Hence population-based varicella incidence and vaccination coverage can not be estimated from sentinel data.

Representative nationwide data on vaccination coverage of children under two years of age are not available in Germany. Yet, they are particularly important because children should be fully immunized at the age of two years. Therefore, the Robert Koch Institute (RKI) initiated a project for the surveillance of vaccine preventable infectious diseases and vaccinations using data from the Associations of Statutory Health Insurance Physicians (ASHIPs) in 2004.

The main objective of our study was to determine varicella vaccination coverage of children under the age of two years in order to investigate the acceptance of the routine varicella vaccination programme in Germany. Furthermore, we aimed to assess if children were vaccinated at the recommended age.

## Methods

### Statutory health insurance data

Physicians who treat statutory health insured persons (approximately 87% of the population) have to report their provided services quarterly to their ASHIP in order to receive payment for their services from Health Insurance Funds. Vaccinations are recorded using specific codes for reimbursement.

Since the establishment of the surveillance project in 2004, ASHIPs send anonymous data on varicella vaccinations to the RKI. Data are not publicly available. Quality assurance of data was done according to the good practice secondary data analysis [14]. During import into a database, data are tested with regard to structure, completeness and plausibility [15]. Data include demographic characteristics of the children (month and year of birth, sex, district of residence), information on the vaccination (type of vaccination, date of vaccination) and information on medical contact (medical specialisation of physician, district of physician's office).

### Analysis

We analysed anonymous data of the years 2004 to 2007 from 13 of 17 ASHIPs: Bavaria, Brandenburg, Bremen, Hamburg, Lower Saxony, Mecklenburg-Western Pomerania, North Rhine, Saarland, Saxony-Anhalt, Saxony, Schleswig-Holstein, Thuringia and Westphalia-Lippe. The regions correspond to 12 of 16 Federal States. Data from Baden-Württemberg, Berlin, Hesse and Rhineland Palatinate were not available for the whole four year period and were therefore excluded from the analysis.

### Study population

The study population consisted of all statutory health insured children under two years of age born in 2004 (cohort 2004) or 2005 (cohort 2005) in one of the 13 above mentioned ASHIPs. The total number of statutory health insured children under two years of age was 415078 in 2004 (85% of all children born in 2004) and 404988 children in 2005 (86% of all children born in 2005) in the studied regions [16-18]. Cohort 2004 was followed-up for 36 months, cohort 2005 for 24 months.

### Varicella vaccination coverage

Vaccination coverage was determined by the proportion of children vaccinated against varicella under two years of age within the study population. For cohort 2004, additionally the vaccination coverage of children under three years of age was determined. A combined measles, mumps, rubella, and varicella (MMRV) vaccine became available in 2006. According to the manufacturer's instructions two doses are required for immunisation, preferably with a minimum of six weeks between the two doses [19]. Children who received one dose of monovalent varicella vaccine or two doses of MMRV vaccine within the first two years of life were defined as vaccinated according to the recommendations of STIKO.

### Statistical analysis

Differences in varicella vaccination coverage between cohort 2004 and cohort 2005 were analysed using uncorrected chi-square statistic. Correlation between level of vaccination coverage and date of introduction of cost coverage for varicella vaccination by Health Insurance Funds was analysed by Spearman's rank correlation coefficient with STATA 10 (StataCorp, Texas, USA).

## Results

### Varicella vaccination coverage

At the age of 24 months, 34% of children of cohort 2004 and 51% of children of cohort 2005 are vaccinated against varicella according to the recommendations of STIKO (Table 1). Vaccination coverage varies greatly between regions. Regarding cohort 2004, the minimum vaccination coverage is found in Bremen (8%) and the maximum in Saxony-Anhalt (69%). Regarding cohort 2005, the minimum vaccination coverage is found in Bremen (21%) and the maximum in Saarland (82%). Children in Federal States of former East Germany have higher varicella vaccination coverage than children in Federal States of former West Germany (p < 0.001 for both cohorts). In most countries, vaccination coverage of cohort 2005 is significantly higher than vaccination coverage of cohort 2004. However in Mecklenburg Western-Pomerania, North Rhine and Saxony-Anhalt vaccination coverage is lower in cohort 2005.

**Table 1:** Varicella vaccination coverage (%) of children under two years of age in Germany

	Vaccination coverage (%)						
	Cohort 2004			Cohort 2005			Difference
Federal State	Varicella vaccine	MMRV vaccine (2 doses)	Total	Varicella vaccine	MMRV vaccine (2 doses)	Total	(p-value*)
Brandenburg	52	<1	52	39	14	53	0.402
Mecklenburg Western-Pomerania	63	<1	63	44	16	60	<0.001
Saxony	29	<1	29	48	<1	48	<0.001
Saxony-Anhalt	69	<1	69	53	11	64	<0.001
Thuringia	45	<1	45	46	3	49	<0.001

East Germany	47	<1	47	46	7	53	<0.001

Bavaria	19	<1	19	43	<1	43	<0.001
Bremen	8	<1	8	20	<1	21	<0.001
Hamburg	35	<1	35	53	8	61	<0.001
Lower Saxony	17	<1	17	46	6	52	<0.001
North Rhine-Westphalia	41	<1	41	48	5	53	<0.001
- North Rhine	52	<1	52	51	<1	51	<0.001
- Westphalia-Lippe	29	<1	29	44	11	55	<0.001
Saarland	48	<1	48	69	13	82	<0.001
Schleswig-Holstein	40	<1	40	37	22	59	<0.001

West Germany	30	<1	30	46	5	51	<0.001

Total	34	<1	34	46	5	51	<0.001

* p-value was calculated using uncorrected chi-square statistic

### Type of varicella vaccine and age of vaccinated children

Within their first two years of life, children of both cohorts were mainly immunised with one dose of varicella monovalent vaccine in the studied regions (Table 2). Around 9% of children of cohort 2005 were vaccinated with two doses of the MMRV vaccine. Vaccinations of both cohorts were mainly given by paediatricians (95%) and general practitioners (4 to 5%). For those children who received two doses of MMRV vaccine, the median time between the doses was 28 weeks for cohort 2004 (inter quartile range (IQR): 7 to 31 weeks) and 15 weeks for cohort 2005 (IQR: 9 to 25 weeks).

**Table 2:** Type of varicella vaccine and number of doses administered to children under two years of age in Germany

	No. of children (%)	
	Cohort 2004	Cohort 2005
Statutory health insured children	415.078 (100)	404.988 (100)
Children vaccinated with varicella monovalent vaccine		
- one dose	138.645 (33)	181.861 (45)
- two doses	1.221 (<1)	4.338 (1)
- more than two doses	18 (<1)	86 (<1)
Children vaccinated with MMRV vaccine		
- one dose	281 (<1)	16.642 (4)
- two doses	95 (<1)	21.718 (5)
- more than two doses	1 (<1)	297 (<1)

**Figure 1:**
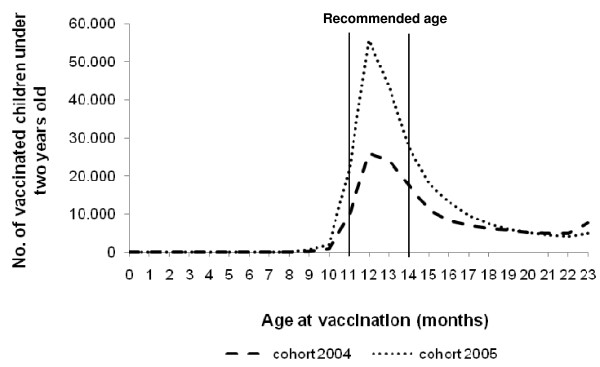
**Age of vaccinated children under two years of age at day of varicella vaccination, cohort 2004 and 2005**.

77159 from 139972 (55%) vaccinated children of cohort 2004 were immunised with the first dose at the recommended age 11 to 14 months. Regarding cohort 2005, 148150 from 224371 (66%) vaccinated children were immunised with the first dose at the recommended age (Figure 1). Children of cohort 2004 had a median age of 14 months at vaccination with monovalent varicella vaccine (IQR: 12 to 17 months) and 23 months for the first dose of MMRV vaccine (IQR: 22 to 23 months). For children of cohort 2005, the median age was 13 months at vaccination with monovalent varicella vaccine (IQR: 12 to 15 months) and 14 months for the first dose of MMRV vaccine (IQR: 12 to 18 months). Analysis of data for cohort 2004 until an age of three years revealed that additionally 50465 children (18% of all unvaccinated children older than the recommended age) were vaccinated between age 24 and 35 months, leading to a vaccination coverage of 46% for children under three years of age.

### Coverage of vaccination costs

**Figure 2:**
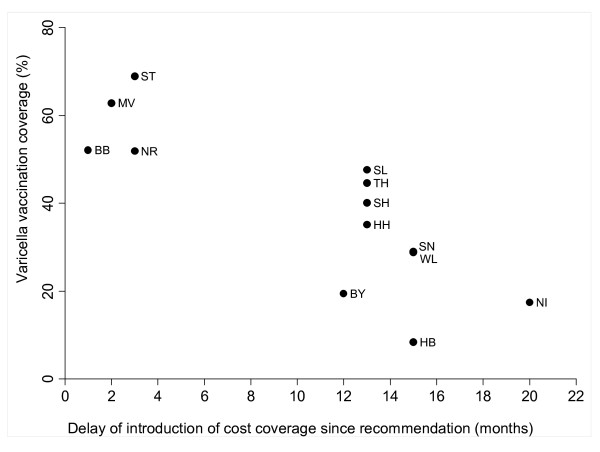
**Varicella vaccination coverage (%) of cohort 2004 in Germany, stratified by Federal State and time points of introduction of cost coverage for varicella vaccination by Health Insurance Funds**.

**Figure 3:**
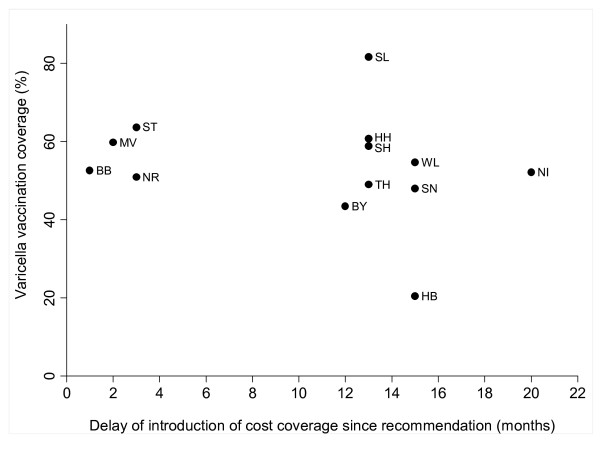
**Varicella vaccination coverage (%) of cohort 2005 in Germany, stratified by Federal State and time points of introduction of cost coverage for varicella vaccination by Health Insurance Funds**.

For cohort 2004, there is a significant association between the level of vaccination coverage and the delay of the introduction of cost coverage since the recommendation of routine varicella vaccination in July 2004 (Spearman's rho = -0.8164; p < 0.001) (Figure 2). The level of vaccination coverage of cohort 2005 and the delay of the introduction of cost coverage were not significantly associated (Spearman's rho = -0.2974; p = 0.324) (Figure 3).

## Discussion

We determined varicella vaccination coverage of statutory health insured under two-year-old children. The results show a significant increase in vaccination coverage from cohort 2004 to cohort 2005. Availability of the tetravalent MMRV vaccine since 2006 might have influenced the acceptance of varicella vaccination. Regional studies in Bavaria and Schleswig-Holstein have also shown an increase in varicella vaccination coverage in young children [11,20]. Population-based incidence of varicella is not available in Germany but data of the varicella sentinel surveillance system showed a steady decrease in the number of varicella cases per reporting physician and month between April 2005 and March 2009. The decrease has been observed in all age groups, in particular for children at 0 to 4 years of age [13].

Regional differences in vaccination coverage could be observed. Children in former Eastern Germany had significantly higher varicella vaccination coverage than children in former Western Germany. Vaccinations were mandatory for school and kindergarten entry in the former German Democratic Republic and therefore a higher acceptance of vaccinations can still be assumed. It has been shown that coverage rates for other vaccinations e.g. for measles are higher in Eastern Germany as well [21]. However, vaccination coverage of children in former Western Germany is increasing [22]. We could show that the level of vaccination coverage of cohort 2004 was significantly associated with the delay in cost coverage since the introduction of the routine varicella programme in July 2004. Our results highlight the importance of vaccinations that are free of charge when aiming for high vaccination coverage. Similar findings were published by the Working Group on Measles and Varicella, showing that sentinel physicians in regions with early cost coverage administered more varicella vaccines than physicians in a region with cost coverage from season 2006/2007 on [13]. International studies have also shown that vaccinations that are free of charge are an important factor in the decision for vaccinations [23].

Another factor that influenced regional differences in vaccination coverage was the delay in the availability of specific codes for reimbursement, even when costs for the varicella vaccine were covered. When routine varicella vaccination was recommended in July 2004, a specific code for the monovalent vaccine has been introduced in North Rhine as late as October 2004. And when the MMRV vaccine became available in July 2006, some ASHIP regions had a specific code for this vaccine only since 2007 (Bremen, Saxony, Saxony-Anhalt, Saarland and Thuringia) or 2008 (Bavaria and North Rhine). Since epidemiological analysis of data is based on these codes, a higher number of children than reported in our study might have been vaccinated in the second half of 2006 and 2007.

More than half of the vaccinated children of cohort 2004 and two thirds of cohort 2005 were immunised at the recommended age by STIKO. The remaining vaccinated children were older than recommended at date of vaccination. This might be explained by catch-up vaccinations in the years after introduction of the routine varicella programme. Our findings are in line with other studies from Germany [21,24]. The percentage of children vaccinated at the recommended age has been constantly increasing since 1996 and the use of combination vaccines seems to contribute to this development [21,25]. The children in cohort 2004 were increasingly vaccinated against varicella at age 24 and 25 months, resulting in a vaccination coverage of 46% at the age of under three years. Around this age, a well-baby check-up is scheduled and it seems that physicians use this check-up for catch-up vaccinations. The median time interval between two doses of MMRV vaccine was 28 weeks for cohort 2004 and 15 weeks for cohort 2005. Thus, physicians followed the manufacturer's instructions regarding the time period of a minimum of six weeks between the two doses.

It has been shown that secondary data from ASHIPS can be used to determine vaccination coverage [15,26]. However, one has to keep in mind that routine health data are primarily collected for reimbursement purposes. Thus, there are limitations in the use of these data when vaccination coverage is determined. In our study, the delay in the availability of specific codes for reimbursement of varicella vaccines had influenced the level of regional vaccination coverage. It is crucial to choose an appropriate denominator for routine health data. In our study, the numbers of statutory health insured children of the birth cohorts 2004 and 2005 were used to determine varicella vaccination coverage. Another valid method is to use contacts between patient and physicians, if known [26]. The data of ASHIPs only relate to statutory health insured children. We assume that coverage of the remaining 14 to 15%, who are mostly privately health insured, is equally high because private health insurances usually cover the costs of recommended vaccinations. We presented varicella vaccination coverage for 12 from 16 German Federal States. All 17 ASHIPs are collaborating partners of the project at RKI. Thus, in future, nationwide data on vaccinations will be available.

## Conclusions

Our study shows increasing varicella vaccination coverage of young children, indicating a growing acceptance of the routine varicella vaccination programme by the parents and physicians. We recommend further monitoring of varicella vaccination coverage using data from ASHIPs to investigate implementation of the routine vaccination programme over time. Data from ASHIPs can also be used for continuous monitoring of other childhood vaccinations that are recommended in Germany.

## List of abbreviations

ASHIPs: Associations of statutory health insurance physicians; BB: Brandenburg; BY: Bavaria; HB: Bremen; HH: Hamburg; IQR: inter quartile range; MMRV: measles, mumps, rubella, varicella; MV: Mecklenburg-Western Pomerania; NI: Lower Saxony; NR: North Rhine; RKI: Robert Koch Institute; SH: Schleswig-Holstein; SL: Saarland; SN: Saxony; ST: Saxony-Anhalt; STIKO: Standing Vaccination Committee; TH: Thuringia; WL: Westphalia-Lippe

## Competing interests

The authors declare that they have no competing interests.

## Authors' contributions

AR carried out the analysis and interpretation of the data, participated in the coordination of the study and drafted the manuscript. MF and LK carried out management of data. AS contributed to the interpretation and revised the manuscript critically. TE and GP participated in the coordination of the study and revised the manuscript critically. All authors read and approved the final manuscript.

## Pre-publication history

The pre-publication history for this paper can be accessed here:

http://www.biomedcentral.com/1471-2458/10/502/prepub
